# Soil-transmitted helminths and associated factors among pregnant women in Doreni district, Oromia region, Ethiopia: a cross-sectional study

**DOI:** 10.1186/s12879-024-09331-y

**Published:** 2024-04-24

**Authors:** Adamu Tesfa Mekonen, Teshome Bekana Hirpha, Asrat Zewdie

**Affiliations:** 1 Doreni Woreda Health Office, Ilu Aba Bor Zone, Mattu, Oromia Region Ethiopia; 2 Department of Medical Laboratory, College of Health Science, Mattu University, Mattu, Oromia Region Ethiopia; 3 Department of Public Health, College of Health Science, Mattu University, Mattu, Oromia Region Ethiopia

**Keywords:** Soil-transmitted helminth, Pregnant women, Ethiopia

## Abstract

**Background:**

Soil-transmitted helminthiasis (STH) refers to a set of parasitic illnesses caused by nematode worms and spread to people through faeces-contaminated soil. It is highly prevalent in low- and middle-income countries due to a lack of environmental sanitation and personal hygiene. Pregnant women are among the risk groups for infection by soil-transmitted helminths. Former studies of the disease burden among pregnant women in Ethiopia didn’t indicate the intensity of parasitic infection. The aim of this study was to assess the prevalence and associated factors of soil-transmitted helminths among pregnant women.

**Methods:**

An institution-based cross-sectional study was conducted among 416 randomly selected pregnant women. The data were collected using a structured interview-administered questionnaire and a laboratory test. The Kato-Katz technique was used to diagnose soil-transmitted helminthiasis and determine the intensity of the infection. The collected data were entered into Epi-Data version 4.6 and exported to SPSS version 25 for analysis. Multivariate logistic regression analysis was used to identify independent predictors of soil-transmitted helminths at a *p*-value < 0.05.

**Results:**

The overall prevalence of soil-transmitted helminths among pregnant women was 30%. (95%, CI: 26-34%). Living in a rural area (AOR = 3.35; 95% CI = 1.83–6.13), drinking from an unprotected water source (AOR = 2.52; 95% CI = 1.45–4.37), not washing one’s hand after the toilet (AOR = 2.75; 95% CI = 1.55–4.88), lacking health information (AOR = 1.70; 95% CI = 1.01–2.85), working as a daily labourer (AOR = 2.88; 95% CI = 1.01–8.20), and walking barefoot (AOR = 4.00; 95% CI = 2.29–7.00) were significantly associated with the presence of soil-transmitted helminths among pregnant women.

**Conclusion:**

The prevalence of STH was significantly moderate in the study area, where pregnant women were mostly affected by ascariasis and hookworms. Living in a rural area, being a daily labourer, walking barefoot, not washing hands after the toilet, drinking from an unprotected water source, and lacking health information were the determining factors. Interventions including health education, the expansion of pure drinking water sources, the promotion of personal hygiene, and the wearing of shoes are recommended to reduce the burden of soil-transmitted helminths in the study area.

**Supplementary Information:**

The online version contains supplementary material available at 10.1186/s12879-024-09331-y.

## Background

Soil-transmitted helminths refer to the intestinal worms that infect humans and spread through contaminated soil [1]. Among the most common parasites on the globe are the three predominant soil-transmitted helminths (STHs), which are *Ascaris lumbricoides*, *Trichuris trichiura*, and *Necator americanus/Ancylostoma duodenale* [2]. Globally, 500 million individuals have trichuriasis, 700–900 million people have hookworms, 800–1000 people are reported to have Ascaris lumbricoides, and 300 million people experience severe morbidity or even death [3]. Additionally, 44 million pregnancies are thought to be affected annually by soil-transmitted helminths (STHs), such as hookworms [4].

A quarter of the world’s population is contaminated with helminths that are spread through the soil. The world’s endemic areas for soil-transmitted helminthiasis are home to 250 million women and girls [5]. Due to the accompanying poverty, severe morbidity, and DALYs lost, STH has been included in the WHO's list of 17 neglected tropical diseases (NTDs) [6]. Pregnant women are particularly sensitive to infection, and the high rates of infection among them are mostly suggestive of faecal pollution of the soil and domestic water supply around homes as a result of inadequate sanitation and incorrect sewage disposal [7].

Sub-Saharan Africa has a highly overlapping geographic distribution of geo-helminthiasis; the concurrent presence of hookworm infection during pregnancy may significantly increase the prevalence of soil-transmitted helminths (STHs) [8]. Soil-transmitted helminths (STHs) are serious public health problems that occur mostly in underdeveloped nations, especially in sub-Saharan Africa [9]. There is a high prevalence of intestinal parasitic infection in Ethiopia, which can be caused by unsafe or insufficient water, unhygienic living conditions, improper latrine use, or the habit of walking barefoot [10]. Despite years of effort to increase the availability of latrine facilities, it is still difficult to find a village that is completely free from open defecation [11].

Ethiopia has implemented the strategy of deworming programmes (only after the first trimester in pregnant women) and vitamin supplementation for the targeted demographics to lessen the burden of STH-associated micronutrient malnutrition, and this framework was useful for planning public health administrations in rural communities in endemic populations [12–14]. Despite the fact that various studies have been conducted and that intervention approaches are used to control and prevent intestinal parasitic infections in Ethiopia, information on the frequency and distribution of intestinal parasites among pregnant women is insufficient and often unavailable [15]. Understanding the magnitude and risk factors for infections helps in the design of locally feasible interventions to reduce the burden of soil-transmitted infections in pregnant women. Therefore, this study was designed to determine the prevalence and associated factors of soil-transmitted helminths among pregnant women.

## Methods

### Study area, design and period

**Fig. 1:**
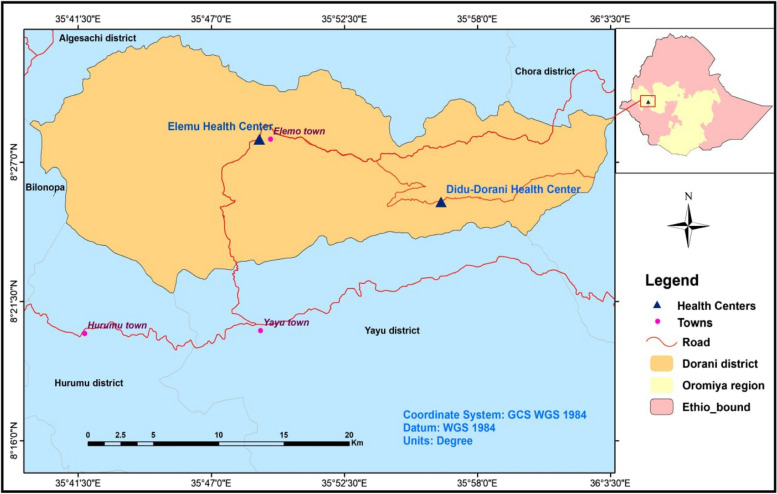
Map of Doreni district administration (the study area), 2023

A health facility-based cross-sectional study was conducted among pregnant women in two public health centres in Doreni district, Ilu Aba Bor zone, southwestern Ethiopia, from May to June 2023. The Doreni district is one of the 14 districts of the Ilu Aba Bor zone. It is located 576 km southwest of Addis Ababa, the capital city of Ethiopia. A district is a local administration containing at least 55,772 people, and it is then divided into kebeles (the lowest administrative level), which contain approximately 3000 people. The people in the area are subsistence farmers who largely rely on animal husbandry and agriculture for their livelihoods. Several water bodies, including perennial rivers, small streams, canals, and rain-filled ponds, are located in the study area. The majority of the community got drinking water from unprotected water sources like rivers and unprotected springs, which accounts for 56.7%. The Doreni district is geographically located between 93′71′02 North latitude and 81′01′28 East longitude, with an elevation of 1990 m above sea level (Fig. 1).

### Sample size determination and sampling procedure

The sample size was calculated using a single proportion formula ${\text{n}}=\frac{{{\text{Z}}}^{2}\left(1-\frac{\mathrm{\alpha }}{2}\right){\text{P}}(1-{\text{P}})}{{{\text{d}}}^{2}}$, where n is the sample size, Z is the statistic corresponding to a 95% confidence interval (1.96), d is the precision of the estimate, and P is the assumed prevalence of soil-transmitted helminths (51.5%) [3]. The sample size was adjusted to 422 by including an additional 38 (10%) pregnant women to compensate for the refusal to submit stool specimens and to reduce errors that may occur during Kato–Katz preparation. The total sample size was allocated to each health center using proportional allocation. Accordingly, 254 pregnant women from the Elemo Health Centre and 168 from the Didu Doreni Health Centre participated in the study. A systematic random sampling technique was used to select pregnant women who were attending antenatal care at the time of data collection. Considering the number of pregnant women who attended ANC in two health centers in the six months prior to the data collection time, the average number of pregnant women expected to attend ANC per month in both health centers was estimated which was 661. Accordingly, the sampling interval (K) was two, and the first participant was selected by lottery method, and then every second woman in every visit was included in the study. Pregnant women who lived in the study area for more than six months and who were attending antenatal care at the time of data collection were included in the study. Pregnant women who were unwilling to respond/refused to bring stool sample were excluded from the study.

### Data collection procedures and laboratory examinations

Data were collected using an interviewer-administered questionnaire developed for this study after reviewing different literatures and guidelines (Supplementary file), and stool samples were collected from each interviewed pregnant woman. Four health workers (1 BSc nurse, 1 BSc MWN, and two lab technicians) were recruited for the data collection process, and two supervisors with a first degree in the laboratory (BSC) and public health were recruited for supervision. The Kato-Katz technique was used to diagnose soil-transmitted helminthiasis and determine the intensity of infection. To estimate the intensity of the infections, the number of eggs counted in Kato-Katz thick smears was multiplied by a factor of 24 to obtain the fecal egg count in units of eggs per gram of stool (EPG). According to the World Health Organization (WHO), the intensity of ascaris infection can be classified as light (1–4,999 EPG), moderate (5,000–49,999 EPG), or heavy (> 50,000 EPG). Hookworm infection was classified as light (1–1,999 EPG), moderate (2,000–3,999 EPG), or heavy (> 4,000 EPG). Trichuriasis infection is classified as light (1–999 EPG), moderate (1,000–9,999 EPG), or heavy (> 10,000 EPG) [16]. To ensure consistency in egg counting, 10% of the examined Kato-Katz smears were re-examined by a senior laboratory technician. Knowledge of pregnant women about soil transmitted helminths was assessed using yes (1) or no (0) responses to eight-item questions about soil transmitted helminths. The median knowledge score was taken as the cut-off point for deciding the level of knowledge based on the sum of the scores. Values above the median were coded as “1” for good knowledge, while values below the median were coded as “0” for poor knowledge.

#### Operational definitions

- Soil-transmitted helminthiasis (STH): A respondent was labeled as having soil-transmitted helminth if eggs or adult worms of any of the ascaris, hook worms, or tricuris worms in a stool sample under a microscope were detected.
- Single infection: infection from one STH species; double infection: infections from two STH species; and triple infection: three STHs diagnosed in one participant.
- Intensity of STH infection: categorized as a light intensity infection, a moderate intensity infection, or a heavy intensity infection based on the number of eggs observed in the stool sample.
- Ascaris infection intensity: less than 4999 eggs per gramme (epg), 5000–49999 epg, and more than 50,000 epg were defined as light, moderate, and heavy intensity infections, respectively.
- Hookworm infection intensity: less than 1999 epg, 2000–3999 epg, and more than 4000 epg were defined as light, moderate, and heavy intensity infections, respectively.
- Trichuria infection intensity: less than 999 epg, 1000–1999 epg, and more than 10,000 epg were defined as light, moderate, and heavy intensity infections, respectively.
- Good knowledge of STH: if respondent’s answers more than median score from knowledge based questions.
- Poor knowledge on STH: when respondents answers less than median score from knowledge based questions.

### Data processing and analysis

The collected data were entered into Epi-data version 4.6 and exported to SPSS version 25 for further analysis. All the questionnaires were checked for completeness after completion by the study participants. Descriptive statistics, including frequencies and proportions, were computed. To identify the associated factors, variables with a *p* value of less than 0.25 in the bivariable analysis were entered into the multivariable logistic regression analysis for further analysis to identify the independent predictors and control confounding factors. The independent variables were tested for multicollinearity by using collinearity diagnostics like variance inflation factor (VIF) and a tolerance test, so that only variables with a VIF less than 5 (a VIF between 1 and 4) or tolerance above 0.25 (from 0.25 to 1) were included in the model.

Finally, to demonstrate the strength of the associations, adjusted odds ratios (AORs) with 95% confidence intervals (CIs) were calculated. Multivariate logistic regression analysis revealed that variables significantly associated with soil-transmitted helminths had p values less than 0.05. The model's fitness was checked by the Hosmer and Lemeshow goodness-of-fit test (*p* value = 0.588).

### Data quality control

Certified clinical nurses were recruited for the collection of the interview-grounded data. The questionnaire was prepared in English and was restated in Afan Oromo to carry out the interview, which was restated in English to assure consistency. Ferocious training was given to the data collectors concerning the objects of the study and the nature of the participants for 2 days prior to data collection. The pretest was conducted at 5% of the sample size (21 pregnant women), and then the necessary corrections were made to the questionnaire before actual data collection. On-point supervision of the data collectors and the data collection process were carried out by the investigators on a daily basis. The data were checked for absoluteness and thickness, edited, and spread on a daily basis. Eventually, the data were gutted after entry into the computer and exported to SPSS version 25 for analysis.

Stool samples were collected, processed, and examined by laboratory technologists. Quality control of the laboratory tests was performed by two laboratory technologists from other health facilities. Concordant findings are taken as the final measure, and discordant findings were re-examined by a senior laboratory technologist recruited from General Hospital. Before a final judgement concerning the specimen was drawn, a senior laboratory technologist re-examined any inconsistent findings and finally his conclusion was used as a final decision on the specimen. Stool samples, survey responses, and laboratory findings were coded before the analysis.

## Results

### Socio-demographic characteristics of the study participants

Four hundred and sixteen (416) participants were involved in the study, for a response rate of 98.6%, and the mean age was 26.72 ± 3.72 years (range from 19 to 35 years). The majority of them (64.9%) were from rural areas, and nearly half (49%) of the participants were aged younger than 29 years. Three hundred and three (72.8%) of the respondents had a primary education. More than half of the women (50.5%) were housewives, and the majority (90.4%) were married (Table 1).

**Table 1:** Socio-demographic characteristics of pregnant women attending ANC in Doreni district, Ilu Aba Bor zone, Oromia region, Ethiopia, August, 2023

**Variable**	**Categories**	**Frequency**	**Percent (%)**
Residence	Rural	270	64.1
	Urban	146	35.1
Age of woman in year	15-19	45	10.8
	20-24	78	18.8
	25-29	157	37.7
	30-49	136	32.7
Occupation	House wife	210	50.5
	Daily labourer	67	16.1
	Merchant	39	9.4
	Student	44	10.6
	Government employee	46	11.1
Marital status	Single	18	4.3
	Divorced	22	5.3
	Married	376	90.4
Educational Status	Non Formal Education	6	1.4
	Primary Education	303	72.8
	Secondary Education	84	20.2
	College and above	23	5.5
Family size	More than five persons	206	49.5
	Less than five Persons	210	50.5
Presence of < 5 year children in the house	No	32	7.7
	Yes	384	92.3
Children wear diaper	No	182	43.8
	Yes	129	31

### Healthcare, environment and lifestyle-related characteristics

More than half (50.7%) of the women did not obtain health information on soil-transmitted helminths during their contact. The mean (SD) haemoglobin (Hb) level was 11.456 ± 0.69624, and the Hb concentration ranged from 8.90 g/dl to 2.80 g/dl. Among the respondents, 154 (37%) had an Hb of 7–11 g/dl and 262 (63%) had moderate anaemia > 11 g/d (Table 2). The majority of pregnant women drank water from an unprotected water source (236; 56.7%); more than half of the respondents did not wash their hands after using the toilet (61.5%). Only 38.5% of women were washing their hands after the toilet; 13.7% of women had washed their hands with soap. More than half of the women were experienced walking on bare feet (54.1%) (Table 3).

**Table 2:** Health service-related characteristics of pregnant women in Doreni District, Ilu Aba Bor Zone, Oromia region, Ethiopia, August 2023 (*n* = 416)

**Variables**	**Categories**	**Frequency**	**Percent (%)**
Number of ANC contact	Below four ANC Contact	351	84.4
	Above Four ANC Contact	65	15.6
Trimester of pregnancy	Third trimester	171	41.1
	Second trimester	147	35.3
	First trimester	98	23.6
Got health information about STH during ANC follow up	No	211	50.7
	Yes	205	49.3
Number of pregnancy	Primigravida	168	39.9
	Multigravida	250	60.1
Hemoglobin result	7–11 g/dl	154	37
	> 11 g/dl	262	63
Deworming history	No	44	10.6
	Yes	372	89.4

*ANC* Antenatal care, *dl* Deciliter

**Table 3:** Environmental and lifestyle factors related to pregnant women attending ANC in Doreni district, Ilu Aba Bor Zone, Oromia region, Ethiopia, August, 2023

**Variables**	**Categories**	**Frequency**	**Percent (%)**
Water source for drinking	Unprotected water source	236	56.7
	Protected water source	180	43.3
Latrine availability	No	53	12.7
	Yes	363	87.3
Hand washing after toilet	No	256	61.5
	Yes	160	38.5
Hand washing with soap	No	359	86.3
	Yes	57	13.7
Walking on bare foot	Yes	225	54.1
	No	191	45.9
Child excreta disposal	Out of the toilet	128	41.3
	In the toilet	182	58.7
Soil eating	Yes	114	27.4
	No	302	72.6
Eating un washed raw vegetables	No	237	57
	Yes	179	43
Cooked meals before eating	Yes	294	70.7
	No	122	29.3

### Knowledge of pregnant women about soil-transmitted helminths

Among the respondents, 264 (63.5%) had not heard about soil-transmitted helminths. The majority (83.9%) of women responded to contaminated soil and water, consumed raw vegetables and fruits as no way of transmitting soil-transmitted helminths (STHs), and 356 (85.6%) responded to diarrhea and abdominal pain, loss of appetite, general malaise, and weakness as no symptoms of soil-transmitted helminths. A total of 42.1% and 57.9% of the pregnant women had good and poor knowledge of STH, respectively (Table 4).

**Table 4:** Knowledge related factors of soil transmitted helminths among pregnant Women attending ANC in Doreni district, Ilu Aba Bor zone, Oromia region, Ethiopia, August, 2023

**Variables**	**Categories**	**Frequency**	**Percent (%)**
Have heard about STHs	No	264	63.5
	Yes	152	36.5
The cause of STHs is parasitic worm infection	No	268	64.4
	Yes	148	35.6
STH is a communicable disease/contagious	No	306	73.6
	Yes	110	26.4
Women knowledge on the ways of transmission of STHs	No	349	83.9
	Yes	67	16.1
Women knowledge on symptoms of STHs	No	356	85.6
	Yes	60	14.4
There is treatment for STHs	No	349	83.9
	Yes	67	16.1
STHs can be prevented	No	307	73.8
	Yes	109	26.2
Pregnant women are at higher risk for STH	No	292	70.2
	Yes	124	29.8
Knowledge score	Good	175	42.1
	Poor	241	57.9

### Magnitude of soil-transmitted helminths among pregnant women

**Fig. 2:**
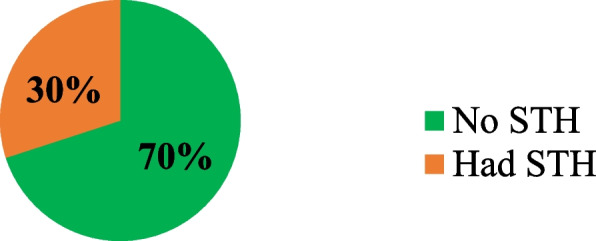
Magnitude of soil-transmitted helminths among pregnant women in Doreni district, Ilu Aba Bor zone, Oromia Ethiopia, August 2023

Soil-transmitted helminths were assessed by stool examinations using the Kato Katz technique. Approximately one hundred twenty-five 125 (30%) (95% CI: 26-34%) participants were identified as having soil-transmitted helminths. Hence, of the identified species Ascaris 77 (61%), Hookworm 33 (26.4%), and Tricuris 9 (7.2%), Ascaris was the dominant species, and 6 (1.4%) of double infections of soil-transmitted helminths were also identified (Fig. 2).

### Stool examination using the Kato Katz technique

Among the total study participants, 125 (30%) were positive for any STH infection. Among the STH species detected, the most prevalent parasite was *A. lumbricoides* (61.6%), followed by hookworms (26.4%) and *T. trichiura* (15%). Double infections with *A. lumbricoides* and hookworm were detected in 6 (4.8%) of the pregnant women’s stool samples. The results of intensity infestations of each STH species showed that Ascaris spurred light-intensity infections, which ranged from 600–2952 EPG (egg per gram) (< 4,999 EPG), and that hook worms had light-intensity infections, which ranged from 672–2592 EPG (< 1,999 EPG) and < 3,999 EPG. Finally, the Trichiura intensity indicated light-intensity infections (528–840) (< 999 EPGs) (Table 5).

**Table 5:** Intensity of soil-transmitted helminth infections among pregnant women attending ANC in Doreni district, Ilu Aba Bor Zone, Oromia region, Ethiopia, 2023 (*n* = 416)

**Variables**	**Categories**	**Frequency**	**Percent (%)**
Ascaris species	No	48	38
	Yes	77	61.6
Hookworm species	No	92	73.4
	Yes	33	26.4
Tricuris	No	116	92.8
	Yes	9	7.2
Double infection	No	119	95.2
	Yes	6	4.8
Soil-transmitted helminths	Yes	125	30
	No	291	70
Ascaris intensity infection	Light intensity infection < 4999 epg	77	100
Hook worm Intensity infection	Light intensity infection < 1,999 epg	23	69.69
	Moderate intensity infection 2000-3999 epg	10	30
Tricuris intensity infection	Light intensity infection < 999 epg	9	100

*Epg* eggs per gram, *STH* soil-transmitted helminths

### Factors associated with soil-transmitted helminths

Bivariable logistic regression was performed to identify factors associated with soil-transmitted helminths among pregnant women. Accordingly, the residence of the respondent, walking barefoot, hand washing, occupational status, availability of latrine, health information, water source for drinking, soil eating, and eating unwashed vegetables were significantly associated with soil-transmitted helminths among pregnant women.

According to the multivariable logistic regression analysis, residence in a rural area, not washing hands, drinking from an unprotected water source, lack of health information, working a daily laborer, and walking barefoot were found to be significantly associated with soil-transmitted helminths among pregnant women. Accordingly, the odds of soil-transmitted helminths among pregnant women who live in rural areas were more than three times greater than those among pregnant women who live in urban areas (AOR = 3.35; 95% CI = 1.83–6.13). Additionally, the odds of soil-transmitted helminths among pregnant women who were not hand-washing after using the toilet were two times greater than those among their counterparts (AOR = 2.75; 95% CI = 1.55–4.88). Similarly, the odds of having soil-transmitted helminths among pregnant women who had experienced walking without shoes were more than three times greater than those among those who had experienced walking with shoes (AOR = 4.00; 95% CI = 2.29–7.00), again, the odds of having soil-transmitted helminths among pregnant women who had drunk from an unprotected water source were more than two times greater than those among those who had drunk from a protected water source (AOR = 2.52; 95% CI = 1.45–4.37). In addition, pregnant women who lacked health information on soil-transmitted helminths during ANC follow-up were about two times more likely to be infected than were those who lacked health information (AOR = 1.70; 95% CI = 1.01-2.85). Furthermore, among pregnant women, daily labourers were about three times more likely to be infected with STH than not being a daily labourer was (AOR = 2.88; 95% CI = 1.01–8.20) (Table 6).

**Table 6:** Bivariate and multivariate analysis of factors associated soil transmitted helminths among pregnant women in Doreni district, Ilu Aba Bor zone, Oromia region, Ethiopia, August, 2023

**Variables**	**Categories**	**Soil-transmitted helminths**		**COR (95% CI)**	**AOR(95%CI)**	***P* value**
		**Yes (%)**	**No (%)**			
Residence	Rural	104 (38.52%)	166 (61.48%)	3.73 (2.21,6.29)	3.35 (1.83,6.13)	< 0.000**
	Urban	21 (14.38%)	125 (85.6%)	1	1	
Occupation	House wife	61 (29%)	149 (70.9%)	1.538 (.782,3.024)	1.10 (0.48, 2.49)	0.82
	Daily labourer	11 (16)	56 (83.5%)	3.205 (1.321,7.78)	2.88 (1.01,8.20)	0.048*
	Merchant	17 (30%)	39 (69.6%)	1.44 (.628,3.320)	1.14 (0.41,3.13)	0.80
	Student	19 (48.7%)	20 (51.2%)	0.663 (.277,1.587)	0.57 (0.19, 1.69)	0.31
	Gov’t employee	17 (38.6%)	27 (61.3%)	1	1	
Water source for drinking	Unprotected source	98 (72%)	138 (58.5%)	4.024 (2.48, 6.5)	2.52 (1.45,4.37)	0.001*
	Protected source	27 (15%)	153 (85%)		1	
Walking on barefoot	Yes	100 (44.4%)	125 (55.6%)	5.3 (3.24,8.72)	4.00 (2.29,7.00)	0.000**
	No	25 (13%)	166 (86.9%)	1	1	
Health information	No	79 (35.26%)	145 (64.7%)	1.73 (1.2, 2.62)	1.70 (1.01,2.85)	0.045*
	Yes	46 (23.95%)	146 (76%)	1	1	
Hand washing after toilet	No	99 (38.67%)	157 (61.3%)	3.24 (1.99, 5.3)	2.75 (1.55,4.88)	0.001*
	Yes	26 (16.25%)	134 (83.75%)	1	1	
Latrine available	No	26 (49.1%)	27 (50.9%)	2.56 (1.43, 4.614)	1.52 (0.76,3.04)	0.24
	Yes	99 (27.2%)	264 (72.7%)	1	1	
Soil eating	Yes	45 (39.47%	69 (60.5%	1.81 (1.149, 2.850	1.5 (0.86, 2.69)	0.15
	No	80 (26.49%)	222 (73.5%)	1		
Eating unwashed vegetables	Yes	69 (34.8%)	129 (61.15%)	1.54 (1.015,2.359)	1.25 (0.74,2.10)	0.40
	No	56 (25.68%)	162 (74.3%)	1	1	

*CI* confidence interval, *COR* Crude odds ratio, *AOR* Adjusted odds ratio

^*^Significant predictors at *p* < 0.05

## Discussion

The prevalence of soil-transmitted helminths among pregnant women attending ANC follow-up in the study area was 30% (95% CI: 26-34%).

These findings are also in line with studies performed in Dembia, northwestern Ethiopia (27.6%) [5]; Shewarobit town, Amhara region (27.7%) [17]; Hosanna town (29.5%) [5]; Felege Hiwot (31.5%) [9]; and Systematic Review and Meta-analysis Ethiopia (29%) [18]. This may be due to the similarity of the socioeconomic characteristics of the respondents. However, these results are greater than those of previous studies conducted in Jimma town (19.7%) [19], East Wollega (24.7%) [20], Kenya (13.8%) [8], and Ghana (14.3%) [21]. Cameroon (13.46%) [22], India (8.34%)[13], Nigeria (12%) [23], Southeast Asia (18%) [24] and Iran (16.43%) [20]. Differences in findings among various studies can be explained by variations in geography, socioeconomic conditions, parasitological examination methods, study population sizes, and awareness of the transmission of STHs.

These values are lower than those of previous studies conducted in the Ethiopian Gilgal gibe dam area (41%) [12]. Wore Ilu northeast Ethiopia (43.5%) [25], Mecha district (70.6%) [10], Tigray region (51.5%) [3], Alefa district in the Amhara region (36.7%) [14], Lalo Kile (43.8%) [26], West Gojjam in the Amhara region (37.3%) [27], the Sidama region (35.8%) [20], and Colombia (41%) [28]. The reason might be due to latrine coverage, geographic location, physical environment, and study populations’ sociodemographic status. The humidity and temperature status of any geographic location affect the viability of most parasites.

According to the results of this study, the odds of soil-transmitted infection were three times greater for pregnant women living in rural areas. These findings are consistent with previous studies performed in Mecha District [10], Yirgalem General Hospital Sidama [20], and Alefa, Amhara [14]. The reason might be due to rural pregnant women's limited access to primary medical services. Additionally, pregnant women in rural areas who frequently work barefoot on contaminated soil are at risk of contracting STH. Additionally, due to their poor environmental and personal hygiene habits, pregnant women residing in rural areas have a high risk of contracting an intestinal parasitic infection.

According to the current study, pregnant women who did not wash their hands after toileting were 2.7 times more likely to have STH than pregnant women who washed their hands after toileting. This finding is supported by studies conducted in Lalo Kile [26], Shewarobit Town in Amhara [17], and Yirgalem General Hospital [20]. This is because proper hand washing practices break the chain of transmission. Similarly, the odds of contracting a soil-transmitted infection were 2.5 times greater among pregnant women who used unprotected water sources for drinking than among those who used protected water sources. This finding is supported by the findings of a previous study at Yirgalem Hospital, Sidama, [20]. Shewarobit in Amhara [17]. Wore Ilu, Northeast Ethiopia [25]; and Alefa district, Amhara [14]. The reason might be that if water supplies are not safeguarded, users may be more likely to be exposed to numerous parasites and thereby to soil-transmitted helminths. The majority of respondents in the study area continued to drink from unprotected water sources, including unprotected springs and rivers.

Moreover, the prevalence of soil-transmitted infections was four times greater among pregnant women who walked on their bare feet than among those who were wearing shoes. This finding is supported by the findings of previous studies conducted in the Lalo Kile District [26], Shewarobit town Amhara [17], and the State of India [13]. This is because soil-transmitted helminths, such as hookworms, are prevented from entering susceptible hosts. These findings also showed that pregnant women who did not obtain health information on soil-transmitted helminths during ANC follow-up were at greater risk than their counterparts were. This finding is also supported by the findings of previous studies performed in the Shoa Zone in Amhara [17] and the West Gojjam Zone [27]. The majority of pregnant women did not obtain health information about their STHs during ANC follow-up, which may be related to the health-seeking behaviour of educated pregnant women.

The present study showed that the odds of obtaining soil-transmitted helminths were greater among pregnant women who were daily labourers than among their counterparts. This finding is supported by a study performed at Shahura Primary Hospital in the Alefa District Amhara region [14]. This is due to the absence of available sanitation facilities around the work area and an environment infected with parasites.

## Conclusions

The prevalence of soil-transmitted helminthiasis (STH) was significantly moderate in the study area, where pregnant mothers were mostly affected by ascariasis and hookworms. The prevalence is moderate according to the Ministry of Health's (MoH) STH endemic area classification. There are three categories: high transmission areas (prevalence rate > 50%), moderate transmission areas (prevalence rate between 20 and 50%), and low transmission areas (less than 20%).The findings of this study showed that living in a rural area, being a daily laborer, walking barefoot, not washing hands after the toilet, drinking from an unprotected water source, and lacking health information were the determining factors. Interventions, including health education, the expansion of pure drinking water sources, the promotion of personal hygiene, and the wearing of shoes by the general population, are recommended to reduce the burden of soil-transmitted helminths in the study area. Besides, preventive chemotherapy (deworming) using single-dose albendazole (400 mg) or mebendazole (500 mg) is recommended as a public health intervention for pregnant women after the first trimester.

### Supplementary Information

**Additional file 1.** Annex I. Annex II.

## Data Availability

The data sets used and/or analysed during this study are available and can be accessed from the corresponding author at any time.

## Abbreviations

ANC: Antenatal care
AOR: Adjusted odds ratio
COR: Crude odds ratio
CI: Confidence interval
EPG: Eggs per gram
ETB: Ethiopian birr
STH: Soil-transmitted helminths
WHO: World Health Organization
