# Supplementary material for: Soil-transmitted helminths and associated factors among pregnant women in Doreni district, Oromia region, Ethiopia: a cross-sectional study
Source: BMC Infect Dis. 2024 Apr 24;24:435. doi: 10.1186/s12879-024-09331-y (PMC11040745; doi:10.1186/s12879-024-09331-y)
Supplement: Supplementary file 1 — Additional file 1. Annex I. Annex II. [file 12879_2024_9331_MOESM1_ESM.docx]

ANNEX I

| **Hosmer and Lemeshow Test** | | | |
| --- | --- | --- | --- |
| Step | Chi-square | Df | Sig. |
| 1 | 6.529 | 8 | .588 |

| **Variables in the Equation** | | | | | | |
| --- | --- | --- | --- | --- | --- | --- |
|  | | B | S.E. | Wald | df | Sig. |
|  |  |  |  |  |  |  |
| Step 1^a^ | Occupation_Cat |  |  | 9.487 | 4 | .050 |
|  | Occupation_Cat(1) | .096 | .417 | .053 | 1 | .818 |
|  | Occupation_Cat(2) | 1.056 | .534 | 3.906 | 1 | .048 |
|  | Occupation_Cat(3) | .128 | .517 | .062 | 1 | .804 |
|  | Occupation_Cat(4) | -.559 | .551 | 1.028 | 1 | .311 |
|  | Residence(1) | 1.210 | .308 | 15.416 | 1 | .000 |
|  | Soil Eating_Cat(1) | .421 | .289 | 2.118 | 1 | .146 |
|  | HandWashing_Cat(1) | 1.011 | .292 | 11.980 | 1 | .001 |
|  | EtaingUnwashed wegitables_Cat(1) | .222 | .265 | .702 | 1 | .402 |
|  | Water Source_Cat(1) | .923 | .281 | 10.774 | 1 | .001 |
|  | Walking on barefoot_Cat(1) | 1.387 | .285 | 23.646 | 1 | .000 |
|  | Latrine availablity(1) | .415 | .355 | 1.369 | 1 | .242 |
|  | Health Informatio(1) | .529 | .264 | 4.025 | 1 | .045 |
|  | Constant | -1.812 | .527 | 11.821 | 1 | .001 |

| **Variables in the Equation** | | | | |
| --- | --- | --- | --- | --- |
|  | | Exp(B) | 95% C.I.for EXP(B) | |
|  |  |  | Lower | Upper |
| Step 1^a^ | Occupation_Cat |  |  |  |
|  | Occupation_Cat(1) | 1.101 | .486 | 2.493 |
|  | Occupation_Cat(2) | 2.875 | 1.009 | 8.194 |
|  | Occupation_Cat(3) | 1.137 | .413 | 3.130 |
|  | Occupation_Cat(4) | .572 | .194 | 1.685 |
|  | Residence(1) | 3.352 | 1.833 | 6.132 |
|  | Soil Eating_Cat(1) | 1.524 | .864 | 2.687 |
|  | HandWashing_Cat(1) | 2.750 | 1.551 | 4.876 |
|  | EtaingUnwashed wegitables_Cat(1) | 1.248 | .743 | 2.097 |
|  | Water Source_Cat(1) | 2.517 | 1.451 | 4.369 |
|  | Walking on barefoot_Cat(1) | 4.004 | 2.289 | 7.004 |
|  | Latrine availablity(1) | 1.515 | .755 | 3.039 |
|  | Health Informatio(1) | 1.697 | 1.012 | 2.846 |
|  | Constant | .163 |  |  |

| a. Variable(s) entered on step 1: Occupation_Cat, Residence, Soil Eating_Cat, HandWashing_Cat, EtaingUnwashed wegitables_Cat, Water Source_Cat, Walking on barefoot_Cat, Latrine availablity, Health Informatio. |
| --- |

# Annex II. English Version Questionnaire

Questions prepared to assess the prevalence of soil-transmitted helminths and its associated factors among pregnant women in Doreni district, Ilu Aba Bor zone, Oromia, Ethiopia.

**Instruction:** Circle on the response which best matches the answer of the respondent.

01. Questionnaire code……………………

02. Client code……………………….

03. Date of data collection: ___/____/_____

**Part one: Socio-demographic characteristics of the participants**

| **Code** | **Questions** | **Response categories** | **Skip** |
| --- | --- | --- | --- |
| S101. | Age (in years) | ____________________________ |  |
| S102 | Marital status | 1. Single 2. Divorced 3. Separated 4. Married |  |
| S103 | Educational status | 1. No formal education 2. Primary education(1–8) 3. Secondary education (9–12) 4. Diploma and above |  |
| S104 | Occupation | 1. House wife 2. Merchant 3. Daily laborer 4. Student 5. Governmental employee 6. Others.____ |  |
| S105 | Residency | 1. Rural 2. Urban |  |
| S106 | Family size (number) | ___________ |  |
| S107 | Family monthly income (in ETB)* | _________________ |  |
| S108 | Is there a <5-year-old child in your house? | 1. Yes 2. No |  |
| S109 | If yes, do children usually wear diapers? | 1. Yes 2. No |  |

**ETB: Ethiopian birr*

**Part two: Health and health related factors**

| **Code** | **Question** | **Response categories** | **Skip** |
| --- | --- | --- | --- |
| H201 | Number of pregnancy | 1. Primigravida 2. Multigravida |  |
| H202 | Status of pregnancy | 1. Wanted 2. Unwanted |  |
| H203 | Trimester of pregnancy | 1. First trimester of pregnancy 2. Second trimester of pregnancy 3. Third trimester of pregnancy |  |
| H204 | Number of ANC contacts | 1. First 2. Second 3. Third 4. Fourth and above |  |
| H205 | History of IP in the previous pregnancy* | 1. Yes 2. No |  |
| H206 | Hgb result (observe from ANC register) | _____________ g/dl |  |
| H207 | Did you get health information on soil-transmitted helminthes during the ANC follow-up? | 1. Yes 2. No |  |
| H208 | Deworming history in the index pregnancy? | 1. Yes 2.No |  |

**ANC: antenatal care IP: Intestinal parasite*

**Part three: Hygiene, environmental sanitation and lifestyle related characteristics**

| **Code** | **Questions** | **Response** | **Skip** |
| --- | --- | --- | --- |
| E301 | Have you experienced soil eating? | 1.Yes 2.No |  |
| E302 | Do you have latrine? | 1.No 2.Yes | If no skip to E305 |
| E303 | If yes, what type of latrine do you have? | 1.Pit latrine  2. Water flushed  3. VIP |  |
| E304 | Are you using your latrine, currently? | 1. No  2. Yes |  |
| E305 | Do you wash your hands after toilet | 1.No  2.Yes | If no skip to E307 |
| E306 | If your answer is “yes “do you use soap for hand washing? | 1. No  2. Yes |  |
| E307 | Where do you dispose child excreta? | 1. Outside toilet  2. In the toilet |  |
| E308 | Have you ever eaten unwashed raw vegetables? | 1. Yes  2. No |  |
| E309 | Do you cook vegetables before eating? | 1. No 2. Yes |  |
| E310 | Do you have experiences of walking on bare foot? | 1.Yes  2. No |  |
| E311 | What is your source of drinking water? | 1. River 2. Pipe line 3. Unprotected Spring 4. Protected Spring 5. Un Protected Well 6. Protected well |  |

**Part four: Knowledge assessment towards soil- transmitted helminthes**

| **Code** | **Questions** | **Response categories** | **Skip** |
| --- | --- | --- | --- |
| K401 | Have you ever heard about STHs**?** | 1. No 2. Yes |  |
| K402 | Is the cause of STH parasitic worm infection? | 1. No 2. Yes |  |
| K403 | Is STH a communicable disease or contagious? | 1. No 2. Yes |  |
| K404 | Can contaminated soil and water and consumption of raw vegetables and fruits transmit STHs? | 1. No 2. Yes |  |
| K405 | Diarrhea, abdominal pain, loss of appetite, general malaise, and weakness are symptoms of STHs. | 1. No 2. Yes |  |
| K406 | Is there a treatment for STHs? | 1. No 2. Yes |  |
| K407 | Can STHs be prevented? | 1. No 2. Yes |  |
| K408 | Are pregnant women at higher risk for STHs? | 1. No 2. Yes |  |

***LABORATORY DATA COLLECTION FORMAT***

| **Code** | **Questions** | **Response** | **Skip** |
| --- | --- | --- | --- |
| L501 | Does the respondents diagnosed with STH | 1.Yes  2.No | If no stop (End) |
| L502 | If yes, is it **Ascaris?** | 1.Yes  2.No | If no, skip to L504 |
| L503 | Ascaris eggs counted | 1. 1-4,999 eggs per gram (EPG)  2.5000- 49,999 EPG  3. >50,000 EPG |  |
| L504 | If yes, is it **Hookworm?** | 1.Yes  2.No | If no, skip to L506 |
| L505 | If yes, is it Ancylostoma duodenale? | 1.Yes  2.No |  |
| L506 | If yes, is it necator Americanos? | 1.Yes  2.No |  |
| L507 | Number of Hookworm egg/larvae counted | 1.1-1,999 EPG  2. 2000-3,999 EPG  3. > 4000 EPG |  |
| L508 | If yes**,** is it **Tricuris?** | 1.Yes  2.No | If no, skip to L510 |
| L509 | If yes, how many Tricuris eggs were counted? | 1.1- 999 EPG  2. 1000-9,999 EPG  3. >10,000 EPG |  |
| L509 | Is there double infection? | 1. Yes 2. No |  |
| L510 | If yes, what is the type of species for a double infection? | 1.Ascaris and Hook worm  2.Ascaris and Tricuris  3. Hook worm and Tricuris |  |
| L511 | Is there a triple infection? | 1. Yes 2. No |  |
